# Comparative Analysis and Characterization of Plastid Genomes of *Mycetia* (Rubiaceae)

**DOI:** 10.3390/genes16121481

**Published:** 2025-12-10

**Authors:** Dongxian Xu, Lingyu Zhang, Chi Zhang, Lei Song, Wanhui Qian, Hao Luo, Qing Zhao

**Affiliations:** Guangdong Academy of Forestry, Guangzhou 510520, China; xudx@sinogaf.cn (D.X.); zly2020@sinogaf.cn (L.Z.); zhangchi@sinogaf.cn (C.Z.); 364875240@sinogaf.cn (L.S.); qianwh@sinogaf.cn (W.Q.); laluohao@sinogaf.cn (H.L.)

**Keywords:** chloroplast genome, *Mycetia hirta*, *Mycetia sinensis*, repeat sequences

## Abstract

Background: *Mycetia*, a subshrub genus within the subfamily Rubioideae (Rubiaceae), is predominantly distributed in tropical Asia, lacking comprehensive plastid genomic resources. This study aimed to characterize the complete plastid genomes of two *Mycetia* species and explore their structural features and evolutionary relationships. Methods: The plastid genomes of *Mycetia hirta* and *Mycetia sinensis* were sequenced and assembled. We analyzed genome structure, simple sequence repeats (SSRs), long repeats, codon usage, nucleotide diversity (π), and Ka/Ks and conducted phylogenetic analysis. Results: Both genomes exhibited a typical quadripartite structure (153,989–154,588 bp; GC content 37.7–37.8%), encoding 126 genes (86 protein-coding, 8 rRNA, and 32 tRNA). Both chloroplast genomes contained 52–60 SSRs and three repeat types with minor interspecific differences. Junction regions and codon usage were highly conserved, with slight variations in RSCU values. The average π was 0.0096, and the non-coding *trnE-trnT* (π = 0.0817) emerged as a potential DNA barcode. The average Ka/Ks was 0.2900, indicating purifying selection. Phylogenetic analysis confirmed the monophyly of *Mycetia* within Argostemmateae. Conclusions: This study provides the first comparative plastid genomic analysis for *Mycetia*, enhancing our understanding of its genetic diversity and supporting future phylogenetic and taxonomic research on the genus.

## 1. Introduction

Rubiaceae Juss. is the fourth largest angiosperm family, comprising 615 genera and 14,266 species [1]. As currently circumscribed, the family is divided into 2 subfamilies (Rubioideae and Cinchonoideae) and 72 tribes, among which the tribe Argostemmateae includes 6 genera, viz., *Argostemma* Wall., *Clarkella* Hook.f., *Leptomischus* Drake, *Mouretia* Pit., *Mycetia* Reinw., and *Neohymenopogon* Bennet [2].

*Mycetia* Reinw. is a moderately sized genus belonging to the tribe Argostemmeae, comprising over 50 species of shrubs or subshrubs distributed predominantly in tropical and subtropical Asia [3]. This genus can be distinguished from other members of the tribe based on the following characters: the bark often is straw-yellow to nearly white, soft, and often corky; calyx lobes are mostly glandular; and the berry-like fruits turn white when mature [4,5]. It is simple to distinguish *Mycetia* from other genera due to berry fruits; however, differentiating among species within this genus remains challenging. Many specimens deposited in herbaria are awaiting identification. Some species in the genus are used as medicine in Asia, such as *M. longifolia* (Wall.) Kuntze and *M. sinensis* (Hemsl.) Craib [6,7,8].

Plastid genome sequences play an important role in interspecific genetic diversity and the reconstruction of plant phylogeny [9,10,11,12,13]. In recent years, complete plastid genomes of numerous angiosperm species have been sequenced and made available in the NCBI database [14,15,16,17,18,19]. Plastid phylogenomic analyses focused on Rubiaceae members have also been conducted extensively in recent years, and have provided significant insights into the evolutionary history of this large family [20,21,22,23,24]. Although Thureborn et al. [23] included two *Mycetia* species (one unidentified) in their study, their work primarily focused on investigating large-scale phylogenetic relationships within the subfamily Rubioideae (Rubiaceae) and exploring the evolutionary characteristics (structure, gene content, and arrangement) of its plastid genome, without detailed examination of the structure of the plastid genome, gene composition, or variation within the genus.

To fill this research gap and additionally select potential molecular markers suitable for species delimitation, this study reports the complete plastid genome sequences of two *Mycetia* species., *M. hirta* Hutch. and *M. sinensis*. Furthermore, we conducted comparative analyses of plastid genome structure between *Mycetia* and its close relatives and performed a plastid phylogenomic analysis of representative Rubiaceae taxa. These results provide fundamental insights into the genomic characteristics of *Mycetia* and establish a reliable genomic resource for future phylogenetic studies.

## 2. Materials and Methods

### 2.1. Plant Material, DNA Extraction, and Sequencing

**Figure 1:**
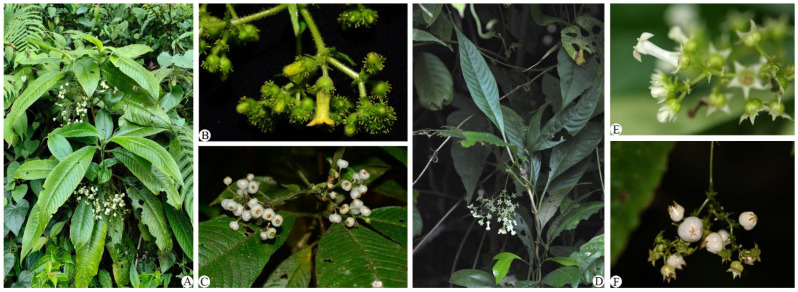
The morphology of *Mycetia hirta* Hutch. ((**A**) habitat, (**B**) inflorescence and flowers, (**C**) fruits) and *Mycetia sinensis* (Hemsl.) Craib ((**D**) habitat, (**B**) inflorescence and (**E**) flowers, (**F**) fruits).

Materials of *M. hirta* and *M. sinensis* were collected on 23 December 2017 from Jinghong City, Yunnan Province, China, and on 31 October 2014 from Napo County, Guangxi Province, China, respectively. The vouchers of the two species are *Z.Q. Song 2017003* and *Napo Exped. 451026141031038*, respectively (Figure 1), and are deposited in the herbaria of the South China Botanical Garden, Chinese Academy of Sciences (IBSC), Guangdong Province, China. Total DNA was extracted from silica-gel-dried leaves using a modified CTAB method [25]. Genomic sequences were obtained through a genome skimming approach [26]. Paired-end (PE) sequencing was conducted on the Illumina HiSeq X-Ten instrument at the Beijing Genomics Institute (BGI) in Wuhan, China.

### 2.2. Assembly and Annotation of Plastid Genome

We employed the GetOrganelle pipeline [27] to assemble the plastome sequence from clean sequencing reads, and Geneious v.9.1.8 [28] and PGA v1.x [29] software were used to verify the accuracy of the assembly and to annotate the plastome obtained.

### 2.3. Data Analysis

#### 2.3.1. Repeat Analysis

MISA software v2.1 [30] (https://webblast.ipk-gatersleben.de/misa (accessed on 20 February 2024)) was utilized to identify simple sequence repeat (SSR) sites in the plastid genome of two *Mycetia* species. Dispersed repeats of four types, i.e., forward repeats (F), reverse repeats (R), complement repeats (C), and palindromic repeats (P), were detected in the plastid genome using REPute v2.5 [31] (https://bibiserv.cebitec.uni-bielefeld.de/reputer (accessed on 26 October 2023)). The parameters were set as follows: minimum length of 30 bp and hamming distance of 3.

#### 2.3.2. IR Junction Analyses

The borders of the inverted repeat (IR) region, large single-copy (LSC) region, and small single-copy (SSC) region junction positions among the *Mycetia* plastome sequences were visualized using IRscope v2.01 (https://irscope.shinyapps.io/irapp/ (accessed on 7 Novermber 2025)) [32].

#### 2.3.3. Relative Condon Usage Analysis

The analysis of relative synonymous codon usage (RSCU) in the plastid genome coding genes was conducted using CodonW v1.4.2 [33] (https://www.softpedia.com/get/Science-CAD/CodonW.shtml (accessed on 25 October 2023)), with default parameter settings.

#### 2.3.4. Genome Comparison Analysis

Homologous gene sequences of different species were compared globally using MAFFT v7.520 [34], and nucleotide variability (π) values were calculated for each gene using DnaSP v6.12.03 [35]. Following this, scatter fold plots were plotted.

#### 2.3.5. Selective Pressure Analysis

Selective pressure analysis was also conducted by calculating the rate of synonymous (Ks) and nonsynonymous (Ka) substitutions and their ratio (Ka/Ks) for all coding gene sequences of the two *Mycetia* species using DnaSP v6.12.03 [35].

#### 2.3.6. Phylogenetic Analysis

Phylogenetic analysis was conducted using 82 plastid coding genes of 3 *Mycetia* species and a further 21 species in Rubiaceae (see Supplement Files Table S5), with *Gentiana delavayi* selected as the outgroup. 16 data were downloaded from NCBI, and 5 data were from Thureborn et al. [23]. The nucleotide sequences were aligned using the plugin of MAFFT v7.520. IQ-TREE v3.0.1 [36] was used to reconstruct the maximum likelihood (ML) tree. According to the Bayesian information criterion (BIC),the best substitution model was recommended with the parameter “-m MFP”; therefore, the TVM+F+R2 model was chosen. To assess the reliability of the phylogenetic tree, the Shimodaira–Hasegawa (SH-aLRT) approximate likelihood ratio test and the ultrafast bootstrap (UFboot) approximation with parameters set at “-alrt 1000 -bb 1000” were used. Clades were considered well-supported if they exhibited an SH-aLRT value of 80% or greater and an UFboot value of 95% or greater.

## 3. Results

### 3.1. Plastid Genome Structure of M. hirta and M. sinensis

**Figure 2:**
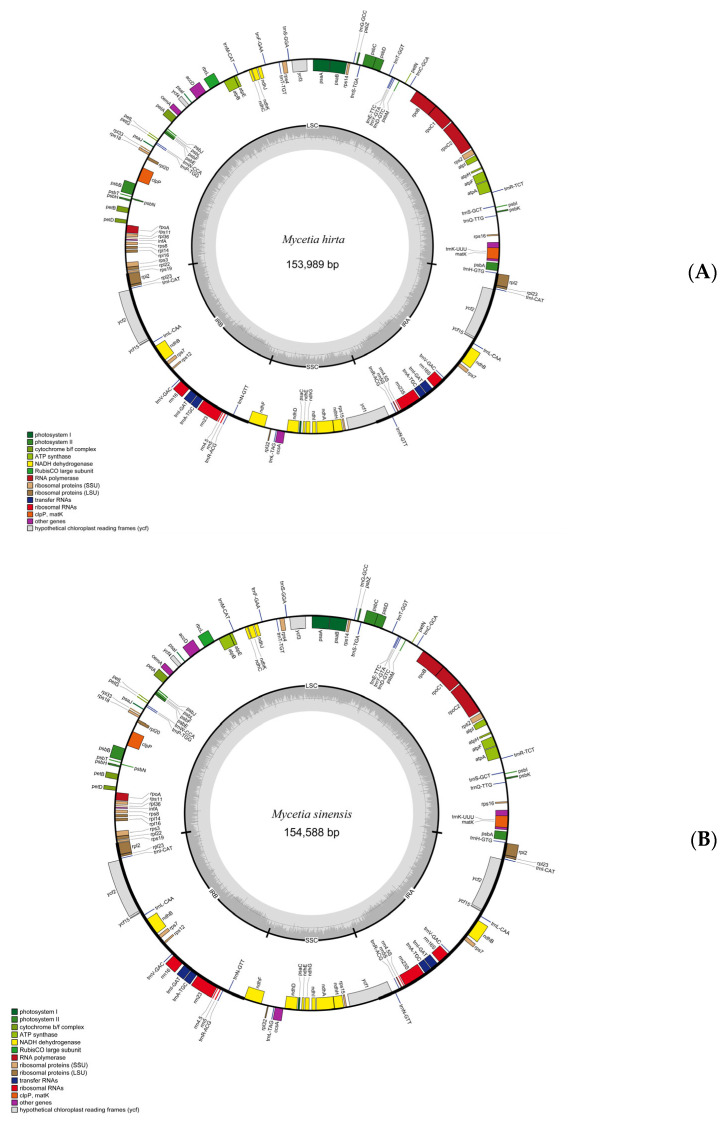
Gene map of *M. hirta* (**A**) and *M. sinensis* (**B**) plastid genomes. LSC and SSC indicate the large and small single-copy regions. IR indicates inverted repeat regions.

The structures of plastid genomes of two species were highly similar. The complete plastid genomes of *M. hirta* and *M. sinensis* are 153,989 bp and 154,588 bp, respectively (Figure 2, Table 1).

The plastid genome structures displayed a typical quadripartite structure with a large single-copy (LSC) region (84,171 bp, 84,736 bp), a small single-copy (SSC) region (17,068 bp, 17,138 bp), and two inverted repeat (IR) regions (52,750 bp, 52,714 bp). The overall GC content was 37.8% in *M. hirta* and 37.7% in *M. sinensis*. In both plastid genomes, the GC contents in the LSC, SSC, and IR regions were 35.6%, 32.3%, and 43.0% in *M. hirta*, and 35.5%, 32.1%, and 43.0% in *M. sinensis*, respectively.

Both plastid genomes contained 126 genes, including 86 CDS genes, 8 rRNAs, and 32 tRNAs. The LSC region contained 61 CDS and 17 tRNA genes, whereas the SSC region comprised 12 CDS genes and only one tRNA gene. Six CDS *(rpl2*, *rpl23*, *ycf2*, *ycf15*, *ndhB* and *rps7*), seven tRNA (*trnI-CAT*, *trnL-CAA*, *trnV-GAC*, *trnI-GAT*, *trnA-TGC*, *trnR-ACG*, *trnN-GT*), and four rRNA *(rrn4.5*, *rrn5*, *rrn16*, and *rrn23*) genes were repeated in the IR regions (Table S1). There were nine genes with introns, of which seven (*ndhA*, *ndhB*, *atpF*, *rpl2*, *rpoC1* and *trnA-TGC*) had only one intron, while the others (*clpP*, *ycf3*) had two (Table S1).

### 3.2. SSR Analysis and Long Repeat Sequences

#### 3.2.1. SSR Analysis

**Figure 3:**
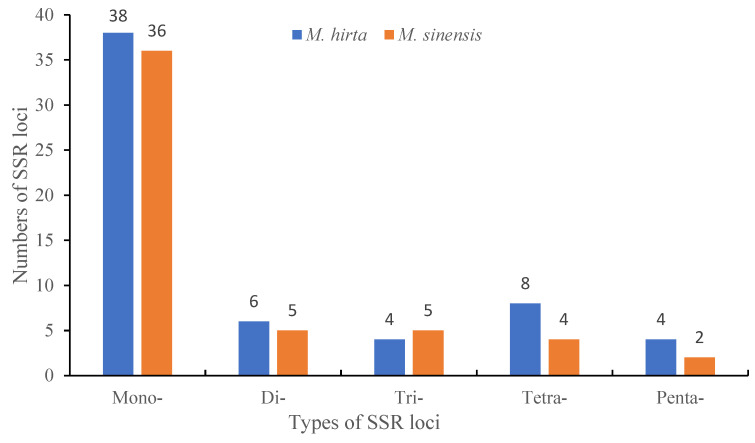
SSR type and number of plastid genomes in *M. hirta* and *M. sinensis.*

SSR microsatellites, also known as simple sequence repeats, were identified in the two *Mycetia* plastid genomes (Figure 3 and Table 2). The number of SSRs discovered in each species ranges from 52 to 60.

Based on SSR analysis, a total of 60 and 52 SSRs were detected in *M. hirta* and *M. sinensis*, respectively. In addition to other plant genes, the most abundant were mono-nucleotide repeats, accounting for 63.3% and 69.2% in *M. hirta* and *M. sinensis*, respectively, followed by di-nucleotides accounting for 10.0% and 9.6%, tri-nucleotides accounting for 6.7% and 9.6%, tetra-nucleotides accounting for 13.3% and 7.7%, and penta-nucleotides accounting for 6.7% and 3.8% in *M. hirta* and *M. sinensis*, respectively. Among the mono-nucleotide repeats, A/T repeats accounted for the total percent.

#### 3.2.2. Long Repeats

**Figure 4:**
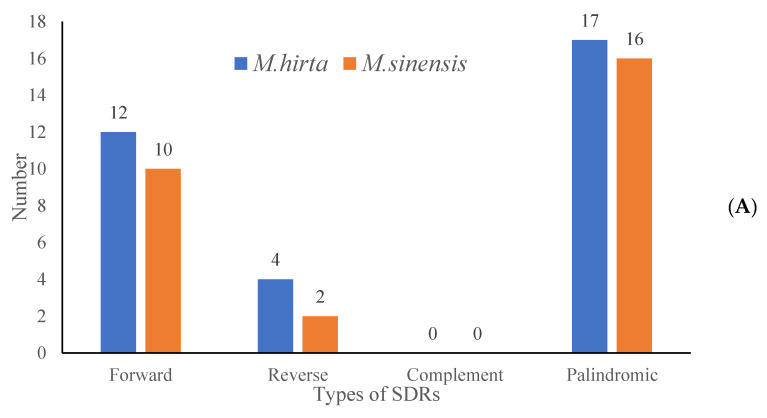

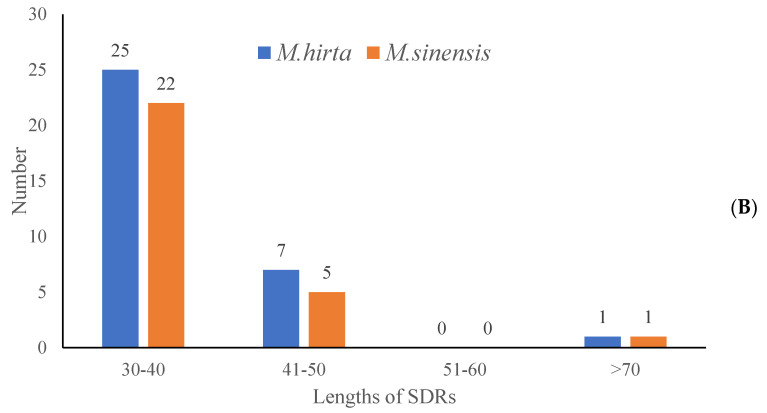
Analysis of short dispersed repeats (SDRs) in *M. hirta* and *M. sinensis*. (**A**) Numbers of four types of SDR; (**B**) numbers of different lengths of SDRs.

**Figure 5:**
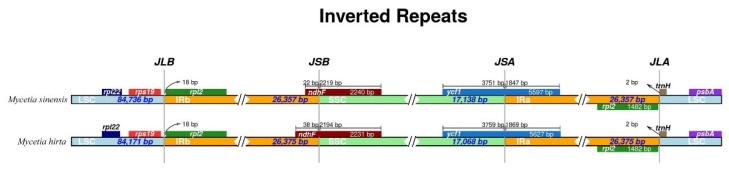
Comparison of the LSC, IR, and SSC borders of *M. hirta* and *M. sinensis*.

To characterize the repeat sequence patterns in the target species, the REPuter software v2.5 was employed for detection, and three repeat types were identified, with detailed distribution results presented in Figure 4A. Specifically, in *M. hirta*, a total of 33 repeat sequences were detected, which could be further categorized into three types (Figure 4B): 12 forward repeats (F), 4 reverse repeats (R), and 17 palindromic repeats (P). For *Mycetia sinensis*, the total number of detected repeats was 28, comprising 10 forward repeats (F), 2 reverse repeats (R), and 16 palindromic repeats (P). Moreover, an analysis of repeat length distribution (as shown in Figure 5) revealed that the majority of these identified repeats fell within the length range of 30–40 base pairs (bp).

### 3.3. IR Expansion and Contraction

The junctions of LSC/IRb, IRb/SSC, SSC/Ira, and IRa/LSC were very similar in the two *Mycetia* species (Figure 5). The *rpsl19* and *rpl2* genes flanked the LSC/IRb (JLB). The IRb/SSC (JSB) and IRa/LSC borders were the *trnN*/*ndhF* and *rpl2/trnH* genes. *NdhF* was entirely found in the SSC region, with lengths of 38 and 22 bp inserted in the IRb/SSC junctions of *M. hirta* and *M. sinensis*, respectively. The *ycf1* gene, crossed the SSC/IRa borders, and was located at the SSC and IRa regions with 3759–3751 bp and 1869–1847 bp lengths in *M. hirta* and *M. sinensis*, respectively.

### 3.4. Codon Usage

**Figure 6:**
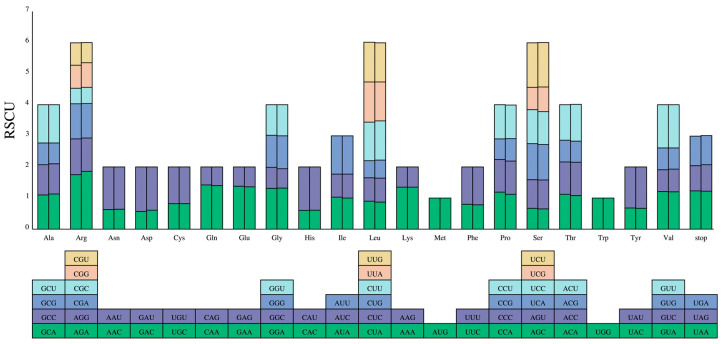
Codon content of 20 amino acids and stop codons in all protein-coding genes of the cp genomes of two *Mycetia* species. Left: *M. hirta*; right: *M. sinensis*. The histogram above each amino acid shows codon usage within *Mycetia*. Colors in the column graph reflected codons in the same colors shown below the figure. RSCU: relative synonymous codon usage.

**Figure 7:**
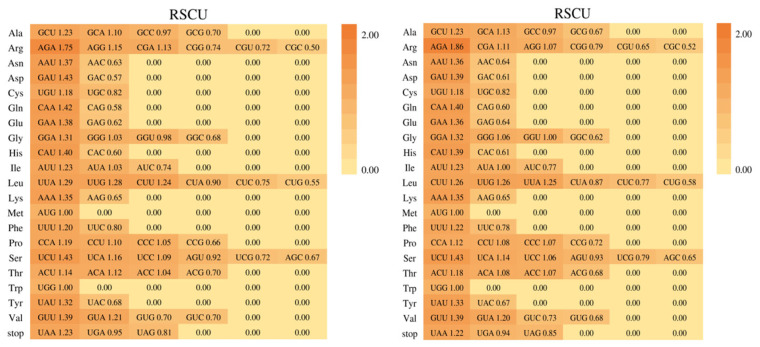
The codon usage of all protein-coding genes of *M. hirta* and *M. sinensis*. **Left**: *M. hirta*; **right**: *M. sinensis*.

The codon usage pattern of the two *Mycetia* species was uniform. A total of 64 codons encoded twenty amino acids. Among the 20 amino acids, methionine (Met) and tryptophan (Trp) were encoded by a single codon, whereas arginine (Arg), leucine (Leu), and serine (Ser) had the maximum codons of six. The relative synonymous codon usage (RSCU) values of the same codon in two species are slightly different (Figure 6 and Figure 7). Both species have 11 high preferences (RSCU > 1.3), 8 moderate preferences (1.2 < RSCU < 1.3), and 14 and 13 low preferences (1.0 < RSCU < 1.2) in *M. hirta* and *M. sinensis*, respectively. Overall, 3, 3, and 13 codons prefer synonymous (RSCU > 1) and C-, G-, and U-ending codons, while 14 (*M. hirta*) and 13 (*M. sinensis*) prefer A-ending codons.

### 3.5. Nucleotide Diversity (Pi)

**Figure 8:**
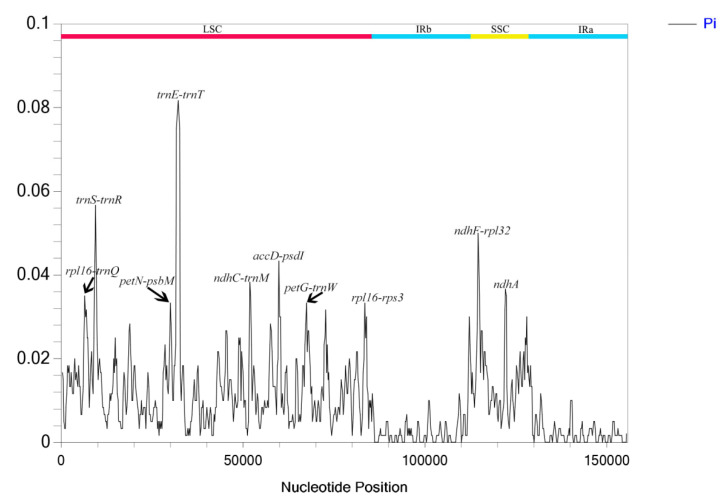
The nucleotide diversity of the plastid genomes of *M. hirta* and *M*. *sinensis*. LSC: large single-copy region; IR: inverted repeat region; SSC small single-copy region.

DnaSP software V. 6.12.03 was used to analyze nucleotide polymorphism in the chloroplast genomes of *M. hirta* and *M. sinensis*. The results showed that their nucleotide variability (π) ranged from 0 to 0.08167, with an average value of 0.0096 (Figure 8). Among them, *trnE-trnT*, *trnT-psbD*, *trnS-trnR*, and *ndhF-rpl32* exhibited relatively high variation, with all π values exceeding 0.02. The highest π value was *trnE-trnT* (0.08167) localized in the LSC region, while most high π values were distributed in the LSC region, and the remainder were located in the SSC region.

### 3.6. Selection Pressure Analysis

**Figure 9:**
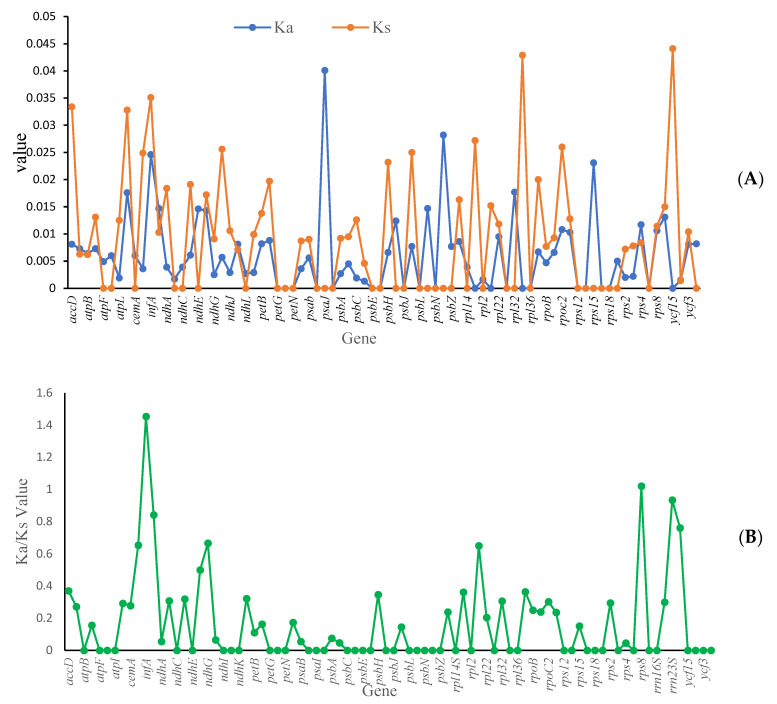
Selective pressure of protein-coding genes in two *Mycetia* species.  (**A**) blue line: Ka, orange line: Ks; (**B**) Ka/Ks ratio. Ka: rate of nonsynonymous substitution. Ks: rate of synonymous substitution.

This study investigated the adaptive evolution of *Mycetia* by analyzing the Ka/Ks ratio. A ratio greater than one indicated positive selection, a ratio less than one indicated purifying selection, and a ratio of one indicated neutral evolution. The average value of Ka was 0.0062, with the highest value of 0.0401 (*psaJ*), and the lowest being 0. The average value of Ks was 0.0090, with the highest value of 0.0441 (*ycf15*) and the lowest being 0 (Figure 9A).

The Ka/Ks ratio reflected selection pressure on genes, and it is a well-recognized marker, which is helpful for understanding the evolutionary forces shaping plastid genes. Generally, genes related to a specific habitat are considered to have undergone strong positive selection; thus, the diversification of habitat type may facilitate an increase in mutation rates. Although the habitats among *Mycetia* vary significantly, synonymous gene mutations occur more regularly than nonsynonymous mutations (Figure 9B). The average value of the Ka/Ks ratio was 0.2900, with the highest value being 1.4272 and the lowest being 0.

### 3.7. Phylogenetic Relationship

**Figure 10:**
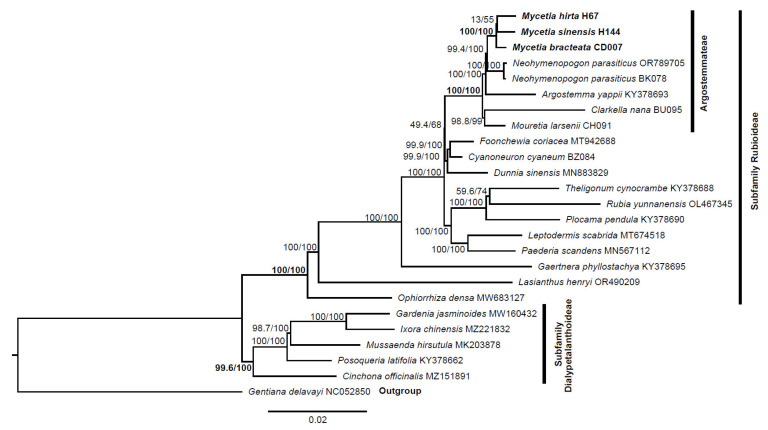
Phylogenetic tree of *Mycetia* species and other species in Rubiaceae based on the alignment of 82 coding genes. Numbers near branches are SH-aLRT and ultrafast bootstrap (UFBoot) support values (i.e., SH-aLRT/UFBoot).

The plastome sequences provide much richer genetic information for revealing phylogeny. We used 82 coding genes to reconstruct phylogenetic trees to identify the phylogenetic position of three *Mycetia* species. The results are presented in Figure 10.

The maximum likelihood (ML) tree reconstructed in this study showed that *Mycetia* species formed a highly robust and well-supported monophyletic clade. This clade was established with maximum bootstrap support values of 100/100, signifying an extremely high confidence level in the grouping of *Mycetia* as a monophyletic unit. The *Mycetia* clade was found to form a sister group with the genus *Neohymenopogon*. When considering the broader taxonomic context, *Mycetia* along with *Argostemma*, *Clarkella*, *Mouretia*, and *Neohymenopogon* jointly constitute the tribe Argostemmae.

On a broad phylogenetic branch, *Foonchewia* and *Cyanoneuron* were observed to form a distinct clade. The *Foonchewia*-*Cyanoneuron* clade formed a sister group with *Dunnia*, and the clade composed of these three genera is sister to the tribe Argostemmateae.

## 4. Discussion

This study presents complete plastid genomes of two *Mycetia* species. Within the Rubiaceae, plastid genome lengths range from 153,093 bp to 160,246 bp [14,23,37,38,39,40,41]. The sequenced *Mycetia* genomes fall within this span, with *M. hirta* at 153,989 bp and *M. sinensis* at 154,588 bp. Consistent with typical plastid genomes, which have a GC content of 30–40%, the overall GC content was 37.8% in *M. hirta* and 37.7% in *M. sinensis*. As expected, the inverted repeat (IR) regions exhibited a higher GC content (43.0% in both species) than the large single-copy (LSC; 35.6% and 35.5%) and small single-copy (SSC; 32.3% and 32.1%) regions. These results indicate that the plastid genome structure and key features of *Mycetia* are consistent with those of other Rubiaceae species.

Significant variation in simple sequence repeat (SSR) abundance is observed across Rubiaceae. For instance, SSR counts in *Uncaria* range from 49 in *Uncaria lancifolia* to 66 in *U. sinensis* [41]. In *Mycetia*, we identified 60 and 52 SSR loci in *M. hirta* and *M. sinensis*, respectively. Mononucleotide repeats were the most abundant type (63.3% in *M. hirta* and 69.2% in *M. sinensis*), and these intraspecific differences may provide valuable information for species delimitation. Congruent with other Rubiaceae taxa, the IR regions contained fewer SSRs than the LSC and SSC regions, and the SSR profile was dominated by mononucleotide A/T repeats. Furthermore, 33 long repeat sequences were detected in the two plastid genomes, categorized into three types. Most repeats were 30–40 bp in length, a characteristic congruent with other Rubiaceae species [38,39,40,41].

Expansion and contraction of the IR regions are common dynamics in plant plastid genomes and directly influence genome size variation. Our analysis of the IR boundaries revealed that there is essentially no variation within the genus *Mycetia*. The two *Mycetia* species are conservative, in agreement with other taxa in the tribe Argostemmateae (Supplement Figure S1), reflecting low interspecific variability in IR region structure within the family.

Codon usage bias, a common feature in most organisms, provides valuable insights into evolutionary mechanisms and patterns. The two sequenced *Mycetia* species exhibited highly consistent codon usage patterns. Of the 64 codons encoding 20 amino acids, methionine (Met) and tryptophan (Trp) were each specified by a single codon, while arginine (Arg), leucine (Leu), and serine (Ser) showed the highest redundancy with 6 codons each. The relative synonymous codon usage (RSCU) values of orthologous codons were similar between the two species. Both species possessed 11 highly preferred codons (RSCU > 1.3) and 8 moderately preferred codons (1.2 < RSCU < 1.3). *M. hirta* had 14 low-preference codons (1.0 < RSCU < 1.2), while *M. sinensis* had 13. Notably, 14 codons in *M. hirta* and 13 in *M. sinensis* showed a preference for A-ending nucleotides.

In plant plastid genomes, nucleotide variability (π) is influenced by core evolutionary processes, namely mutation, selection, genetic drift, and gene flow, as well as biological and environmental factors [42,43,44]. In this study, π values for *Mycetia* ranged from 0 to 0.08167, with a mean of 0.0096. This level of interspecific divergence is typical for closely related species within angiosperms, indicating moderate chloroplast genome differentiation between *M. hirta* and *M. sinensis*. The extreme values highlight the mosaic pattern of evolution in plastid genomes, with highly conserved regions under strong purifying selection (π ≈ 0) alongside hypervariable hotspots (π > 0.08), such as the *trnE-trnT* intergenic spacer.

Variation hotspots were primarily localized in non-coding regions, indicating relatively high nucleotide diversity in these areas. This uneven distribution of variability makes these regions particularly useful for investigating plant evolutionary processes and informing biodiversity conservation.

Phylogenetic analysis confirmed that the two *Mycetia* species with *Mycetia bracteata* in Thureborn et al. [23] form a well-supported monophyletic clade. This result reinforced the taxonomic integrity and evolutionary independence of the genus. Based on our taxon sampling, *Mycetia* and *Neohymenopogon* form a strongly supported sister clade, which aligns with the findings of Thureborn et al. [23]; however, it conflicts with their former work [45]. Such topological inconsistency points to nuclear-cytoplasmic discordance, which underscoring the necessity of integrating multi-locus nuclear data or expanded taxon sampling to resolve the phylogenetic placement of *Mycetia* and identify its authentic sister group. Within the large phylogenetic branch, the genus was nested within the tribe Argostemateae, with four other genera (*Argostemma*, *Clarkella*, *Mouretia*, and *Neohymenopogon*), and this result is also consistent with prior systematic molecular studies [2,23,44,45,46,47]. The formation of this tribe is based on a suite of shared morphological and molecular traits that have been identified through detailed taxonomic and phylogenetic studies [44,45]. Furthermore, our analysis recovered a strongly supported clade comprising the monotypic genera *Foonchewia* and *Cyanoneuron*, which is a sister to the genus *Dunnia*. This topology aligns with earlier phylogenetic research [2,47,48], providing additional support for this evolutionary relationship.

## 5. Conclusions

This study presents the complete plastid genomes of *M. hirta* and *M. sinensis*. Both plastomes exhibit the canonical quadripartite structure of angiosperms, a conserved gene repertoire (127 genes), and highly similar GC content (37.7–37.8%), reflecting strong structural conservation within the genus. Despite this structural conservation, analyses of nucleotide diversity (π) and selective pressure uncovered regions of significant divergence. Notably, the *trnE-trnT* intergenic spacer (a π hotspot) and genes such as *psaJ* and *ycf15* exhibited higher evolutionary rates, marking them promising candidates for developing DNA barcodes. Predominantly low Ka/Ks ratios (mean = 0.2900) indicate widespread purifying selection. Phylogenomic results showed that *Mycetia* is monophyletic and robustly placed *Mycetia* within the tribe Argostemmateae, which is sister to the genus *Neohymenopogon*, supporting its recent taxonomic delineation. Collectively, these findings establish a foundational plastome framework for *Mycetia*, deliver practical molecular markers for population studies, and enhance the genomic resources available for Rubiaceae systematics and conservation.

## Figures and Tables

**Table 1:** The characteristics of *Mycetia hirta* and *Mycetia sinensis* plastomes.

Species	*M. hirta*	*M. sinensis*
Cp genome size (bp)	153,989	154,588
IR (bp)	2750	52,714
LSC (bp)	84,171	84,736
SSC (bp)	17,068	17,138
Total number of genes	126	126
rRNA	8	8
tRNA	32	32
Protein-coding genes	86	86
Overall GC content %	37.8%	37.7%

**Table 2:** Analysis of simple sequence repeats (SSRs) in *M. hirta* and *M. sinensis* plastid genomes.

SSR Type	Repeat Unit	*M. hirta*/Ratio	*M. sinensis*/Ratio
Mono	A/T	38/63.3	36/69.2
Di	AT/AT	6/10.0	5/9.6
Tri	AAC/GTT	1/6.7	1/9.6
	AAG/CTT	0	1
	AAT/ATT	3	3
Tetra	AAAC/GTTT	2/13.3	2/7.7
	AAAG/CTTT	1	1
	AAAT/ATTT	1	0
	AATG/ATTC	1	0
	AATT/AATT	1	0
	ACAT/ATGT	1	1
	ACCG/CGGT	1	0
Penta	AAAAG/CTTTT	1/6.7	0/3.8
	AAATT/AATTT	1	0
	AATAT/ATATT	1	1
	AATCT/AGATT	1	1
	Total	60	52

## Data Availability

The original contributions presented in this study are included in the Supplementary Materials. Further inquiries can be directed to the corresponding author.

## Supplementary Materials

The following supporting information can be downloaded at https://www.mdpi.com/article/10.3390/genes16121481/s1. Table S1 is the plastid genome function; Table S2 is SDR; Table S3 is RSCU; Table S4 is Ka and Ks; Table S5 is detailed species information for phylogenetic tree construction; Figure S1 is the whole *Mycetia* cp genome; Text S1 is *M. hirta* in GFF format; Text S2 is *M. sinensis* in GFF format.
