# Supplementary figures and images for: Comparative Analysis and Characterization of Plastid Genomes of Mycetia (Rubiaceae)
Source: Genes (Basel). 2025 Dec 10;16(12):1481. doi: 10.3390/genes16121481 (PMC12733169; doi:10.3390/genes16121481)

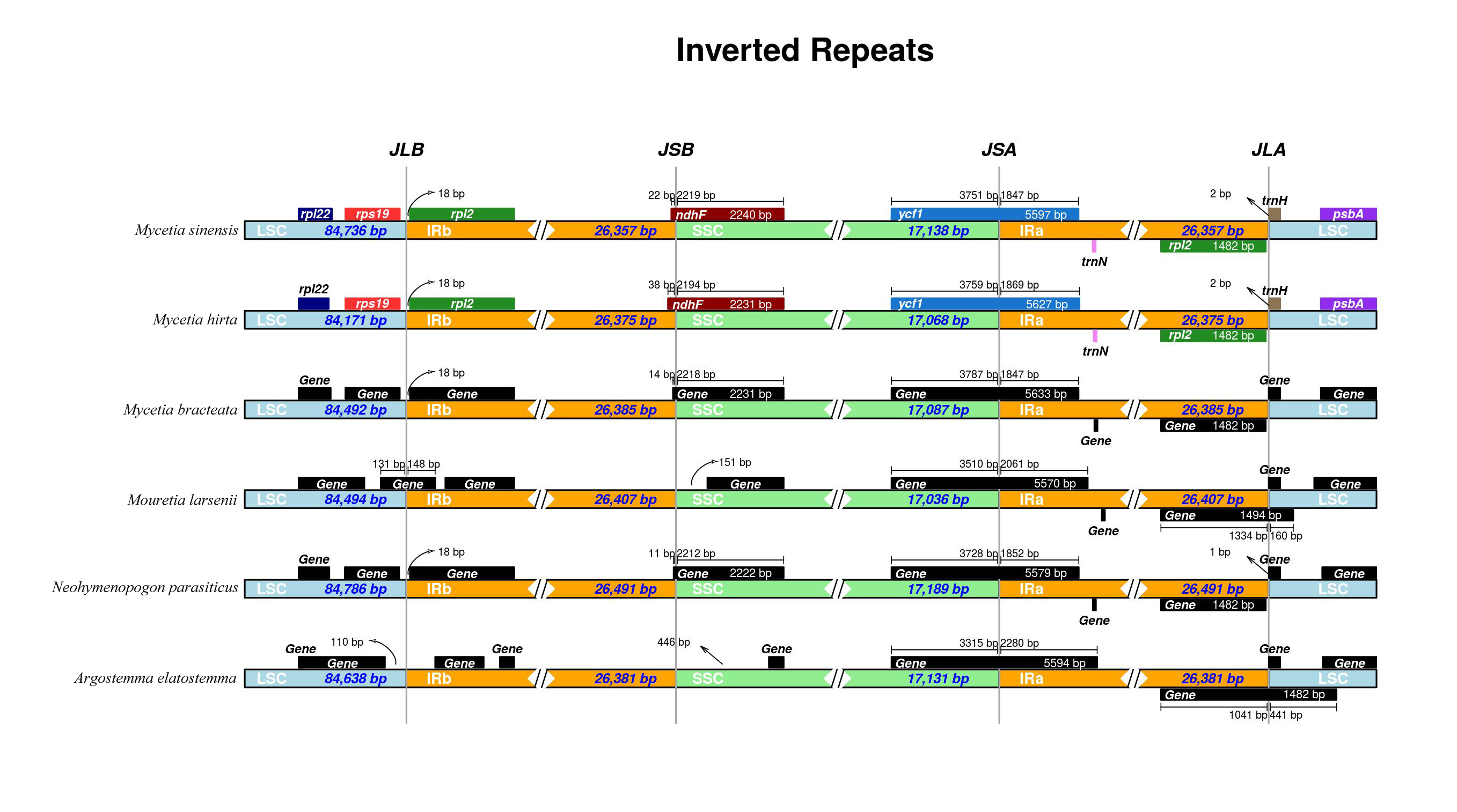

Supplement: Supplementary file 1 [file genes-16-01481-s001.zip › Figure S1.jpg]
